# Healthcare professionals' perspectives on the reproductive management of young women with breast cancer: A qualitative study based on social-ecological system theory

**DOI:** 10.1016/j.apjon.2025.100686

**Published:** 2025-03-11

**Authors:** Jiajia Qiu, Jing Li, Lichen Tang, Ping Li, Mingxuan Cai, Chenxi Zhu

**Affiliations:** a Department of Nursing Administration, Shanghai Cancer Center, Fudan University, Shanghai, China; b Department of Oncology, Shanghai Medical College, Fudan University, Shanghai, China; c Department of Breast Surgery, Shanghai Cancer Center, Fudan University, Shanghai, China; d School of Nursing, Fudan University, Shanghai, China

**Keywords:** Breast cancer, Healthcare professional, Reproductive management, Qualitative research, Social-ecological systems

## Abstract

**Objective:**

To explore the perspectives of healthcare professionals involved in the reproductive management of young women with breast cancer based on social-ecological systems.

**Methods:**

A descriptive qualitative study was conducted. Purposive sampling was used to select 15 healthcare professionals from breast cancer specialties between January and June 2024. Face-to-face in-depth interviews were conducted. Interview data were analyzed using Colaizzi's content analysis method.

**Results:**

Three themes emerged from the data: the reproductive decision-making process needs to involve multiple parties; multiple supports should be provided during reproductive management; and reproductive management requires multi-system preparedness.

**Conclusions:**

From the patient's perspective, we should respect their will, consider family decisions, and strengthen learning abilities. From the professionals' perspective, we should improve the reproductive management awareness of healthcare professionals, their professional learning ability and their cancer reproductive consultation and communication abilities. At the organizational level, social resources could be integrated to provide multidisciplinary information and multi-dimensional emotional support to promote smooth reproductive decision-making in young patients with breast cancer to improve their quality of life.

## Introduction

Breast cancer is among the malignant tumors with the highest incidence rate worldwide. In 2022, there were 2.3 million new patients with breast cancer globally, and 350,000 new patients in China.^1^, ^2^, ^3^ The median age of incidence in China is lower than that in Europe and the United States.^4^ According to the registration data of domestic medical institutions, the proportion of patients with breast cancer aged ≤ 40 years has increased in recent years.^5^ Using data from the Affiliated Cancer Hospital of Fudan University as an example, 7.6% of female patients were < 35 years old, and 23.2% were 35–44 years old.^6^ With the continuous advancement of comprehensive treatments such as surgery, chemotherapy, radiotherapy and endocrine therapy, the survival rate of young patients has significantly improved; however, these treatments cause near- and long-term adverse effects, including damage to ovarian function. Economic development, social progress, liberalization of the two- and three-child policies and the postponement of childbearing have led to more unmarried patients at the time of diagnosis or those who plan to have another child.^7^ Data show that the pregnancy rate in young patients with breast cancer is only 3%, which is 40% lower than that in healthy young women.^8^

Reproductive management of young women with breast cancer in China started recently and has focused mainly on fertility preservation. Most studies have focused on understanding the physiological and psychological indicators related to fertility from the patient's point of view. Patients expressed that the lack of information about fertility and the inability to obtain timely and professional fertility guidance are the main reasons why they believe that fertility and survival cannot coexist.^9^ However, younger patients have a greater demand for reproductive information^10^ and more sensitive psychological characteristics,^11^^,^^12^ which poses more challenges for healthcare professionals in managing their reproductive issues.

However, few studies have explored the reproductive management process from the perspective of healthcare professionals. Clinical staff's concerns about reproductive issues, lack of awareness of fertility protection measures, and lack of access to referrals and other processes may be the main obstacles.^13^ Our research team's previous studies revealed that young women with breast cancer have greater reproductive concerns,^14^ their reproductive decision-making process is affected by various factors, they have various emotional experiences and they exhibit multiple support needs during the reproductive process.^15^ Whether healthcare professionals' perceptions of young patients' reproductive issues are consistent with their needs remains unknown. We focused on healthcare professionals' perspective on fertility preservation and their broader experience of reproductive management.

The social-ecological system theory considers the whole social environment as a kind of social-ecological system and emphasizes the interaction between individuals and the subsystems in the ecosystem. It focuses on the individual as well as family, community, socio-cultural and other related social-ecological systems, and emphasizes the systematic study and analysis of the negative and positive adaptation processes accompanying stress from the level of multifaceted, multilevel, dynamic, and interactive interactions.^16^ According to the theory of social-ecological systems, “the interconnection between individual development and the surrounding environment constitutes a number of systems, i.e., micro-systems, meso-systems, and macro-systems”.^16^^,^^17^ The microsystem is the system of individuals in their social environment, which mainly consists of physiological and psychological factors; the mesosystem is the system of small-scale groups that affect individuals, including families, occupational groups, and other social groups; and the macrosystem is the system of societies that are larger than the small-scale groups, which mainly consist of organizations, societies, and cultures. In order to survive and develop, individuals should interact effectively with their environment, and these three systems are not independent of each other, but influence and interact with each other.^16^

Therefore, this study aimed to explore healthcare management professionals' perspectives on the reproductive management of young women with breast cancer via social-ecological systems at the macro-, meso-, and micro-levels and to provide a scientific, theoretical, and practical basis for the reproductive management of young patients.

## Methods

### Study design

A descriptive qualitative study was conducted to explore the experiences of healthcare professionals during the reproductive management of young women with breast cancer using social-ecological systems. Descriptive qualitative studies aim to obtain the perspectives of the participants on a specific topic directly, and describe the individual's first-hand expressed

Experiences using the content analysis method, which can provide effective information for clinical intervention, grading, needs assessment, questionnaire development and investigation.^18^^,^^19^

### Setting and sample

From January to June 2024, healthcare professionals in the department of breast surgery of a tertiary-level A-class specialized cancer center in Shanghai were selected for the study. Participants were approached individually and recruited by the corresponding author, a nurse with a master's degree, using purposive sampling. The inclusion criteria were: (1) healthcare professionals involved in breast cancer diagnosis, treatment and care and (2) willingness to participate in this study. All the study participants signed an informed consent form. The sample size was determined based on the saturation of the data. In descriptive qualitative research, content analysis requires the researcher to conduct initial coding immediately after each interview. When analyzing the 15th participant, the results began to converge, we found no new themes or information from the 15th participant's interview, so no more participants were recruited.

### Development of interview outline

The interview outline was developed based on the social-ecological system theory. Incorporating the purpose of this study, the literature review, and the results of the research team's previous studies, we developed a draft of the interview outline. Pre-interviews were conducted with two healthcare professionals using this draft. After the pre-interview and a team discussion, the outline was adjusted and modified. Finally, a formal interview was conducted (Table 1).

**Table 1:** Interview outline.

Interview outline
1. How do you think the decision-making process of young patients' fertility is mainly determined at present?
2. What factors play an important role in the reproductive management of young patients?
3. What role do you think patients play in the reproductive decision-making process?
4. What role do you think patients' family members play in the reproductive decision-making process, and what kind of support and help do they provide?
5. What role do you think healthcare professionals should play in patients' reproductive management, and what kind of support and help should they provide?
6. What role do you think social organizations should play, and what kind of support and help should they provide in patients' reproductive management?
7. What do you think should be the form and content of the participation of doctors, other personnel, and patients in reproductive management?
8. What kind of training should doctors and patients receive in the reproductive management of young patients?

### Data collection

The researcher contacted the interviewees to discuss and select an appropriate time and place for the interview. Before the interviews, the purpose, methodology, and content of the study were explained to the participants, their consent was obtained, and an informed consent form was signed. The interviews were conducted according to a semi-structured interview outline, that was appropriately adjusted according to each situation. During the interviews, techniques such as responding, repeating, pursuing and summarizing were used to ensure that the information obtained was as comprehensive, accurate and truthful as possible to reflect the respondents' viewpoints. Recordings and field notes were documented during the interviews. The duration of the interview was 30–60 minutes. The recordings were transcribed into textual materials within 24 ​h of the interviews.

### Data analysis

Descriptive qualitative study uses content analysis to describe the expressed, superficial, and overt experiences of individuals. Content analysis can systematically and objectively analyze unstructured or semi-structured data (such as text, interview) to extract key themes or patterns. In this study, the audio-recorded interviews were transcribed and organized via content analysis method. The researcher conducted initial coding immediately after each interview. As coding begins, statements were categorized based on similar phrases, emotions, experiences, and values to identify themes. Ultimately, common perspectives, experiences, or issues were recognized. Once themes were fully generated, the researcher ensured descriptive validity (precise descriptions conveyed by participants) and interpretive validity (accurate portrayal of participants' expressions about the phenomenon). This process was completed through joint review by the interviewer and interviewee.^18^^,^^20^ The collected data were coded, categorized, and analyzed using NVivo 11.0. Data collection and analysis were performed in parallel. Two researchers worked together to analyze the data, after which the results of the textual analysis were corrected and reviewed by the subject team to ensure date accuracy and precision.

### Rigor

This study strictly followed the standards for reporting qualitative research.^21^ According to our theoretical framework, an interview outline was developed based on a literature review and results of previous studies. An audio recorder was used to record the interviews. Interviews and data analyses were conducted simultaneously. Data saturation was strictly followed to ensure the trustworthiness of the study. The primary researchers were nurses with a master's degrees engaged in long-term clinical work and scientific research. Data analysis was performed independently by two researchers, and their findings were compared. Finally, the research team reviewed themes and reached a consensus. Two participants were invited to discuss the results to ensure that the findings were clear and understandable.

## Results

Fifteen healthcare professionals participated in the study, including three males and 12 females. The mean age was 39.7 years, range: 34–45 years. Most participants had a bachelor's degree or above. The average duration of specialized work was 14.7 years, range: 6–22 years. The detailed characteristics of the participants are represented in Table 2.

**Table 2:** Detailed characteristics of participants (*N* ​= ​15).

No.	Sex	Age, years	Duties	Educational attainment	Years of specialized work	Marital status
N1	Male	39	Surgeon	Doctor	6	Married and having children
N2	Female	44	Surgeon	Doctor	14	Married and having children
N3	Male	43	Surgeon	Doctor	11	Married and having children
N4	Male	45	Surgeon	Doctor	15	Married and having children
N5	Female	39	Head nurse	Bachelor	17	Married and having children
N6	Female	41	Head nurse	Master	19	Married and having children
N7	Female	34	Head nurse	Master	9	Married and having children
N8	Female	39	Head nurse	Bachelor	17	Unmarried and infertile
N9	Female	39	Nurse	Bachelor	17	Married and having children
N10	Female	45	Head nurse	Bachelor	15	Unmarried and infertile
N11	Female	36	Nurse	Bachelor	15	Married and having children
N12	Female	36	Nurse	Bachelor	14	Married and having children
N13	Female	36	Nurse	Bachelor	15	Married and having children
N14	Female	42	Nurse	Junior college	22	Married and having children
N15	Female	37	Nurse	Bachelor	15	Married and having children

Based on the results of the qualitative analysis, eight subthemes emerged from the three main themes summarized in Table 3. Detailed reports on the three themes with supportive descriptive examples follow. All quotations from participants are in italics. Aliases (N1 to 15) were used for each participant.

**Table 3:**
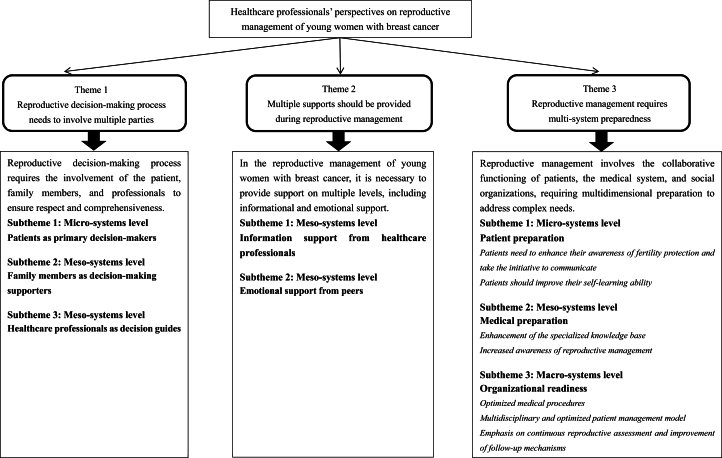
Three themes, eight subthemes and key codes developed in this study.

## Theme 1: Reproductive decision-making process needs to involve multiple parties

Reproductive decision-making process requires the involvement of the patient, family members, and professionals to ensure respect and comprehensiveness.

### Subtheme 1: Micro-systems level-patients as primary decision-makers

Women take the lead in decisions about their own fertility. The patient's decision-making process is influenced by the level of cognition, age, marital and childbearing history, and economic status. N3: *“Women are not saying that it is a one-dimensional thing like in the past when men dominated it and all listened to them, or they have some ideas of their own, and are more rational about it …..."* N11: *“The patient's word is that it should be kind of a major one, or she decides."*

### Subtheme 2: Meso-systems level-family members as decision-making supporters

The attitude of the partner, as a participant in the reproductive decision-making process, greatly affects young patients. N15: *“If her husband can encourage her a little bit, she may have more confidence in doing these things.”* Moreover, partners should not only consider reproduction while ignoring the patient's health and psychological state when discussing fertility. N10: *“He is certain to take the initiative to go forward, but how to take the initiative, the men need to have some wisdom to go to discuss with the woman, not even just with the woman but also with the woman's family. ”*

Parents often put the patient's health first, and when the patient's physiological and economic conditions allow, parents often respect the patient's wishes and support her decisions. N9: *“Parents are supportive of these young patients,* support *her to freeze eggs and these things, whether she will get married or not in the future, I think that their family members are supportive of the attitude.”* N6: *“On the one hand, emotional* support*, on the other hand,* financial support*, this is a very important key role in the fertility decision.”*

### Subtheme 3: Meso-systems level-healthcare professionals as decision guides

Healthcare professionals are often the main source of information, and play a guiding role. N11: *"She's definitely going to ask you which one I'm suitable for, then you're definitely going to list this ABCD program for her first, then at least we have these options right.”* N1: *“The overall guide …... is in the spirit of I'm going to tell you everything, and if she has more understanding, we will go back and talk to her in more detail, and maybe go back and help her with some of the decision-making.”* Moreover, healthcare professionals' understanding of the reality of the patients' conditions and their attitudes and views greatly affect patients' decision-making. N9: *“This time the oncologist's attitude is very important.”* N10: *“I think the key thing is still how our professionals themselves look at fertility, which actually affects our patients directly.”* N15: *“If the doctor tells her that the risk of you having a baby is very small, it will not affect your condition, and then your condition will not be passed on to your child or whatnot, the probability of (agreeing to it) is definitely high."*

## Theme 2: Multiple supports should be provided during reproductive management

In the reproductive management of young women with breast cancer, it is necessary to provide support on multiple levels, including informational and emotional support.

### Subtheme 1: Meso-systems level-information support from healthcare professionals

When patients are diagnosed, they are often anxious, and their ability to accept information is poor. They focus on disease treatment and tend to neglect fertility problems. Among reproductive-aged patients, those with breast cancer have a lower understanding of disease-related reproductive information but a greater demand for it. Therefore, healthcare professionals should comprehensively assess patient's conditions and promptly inform them of options to facilitate patients' early decision-making and early intervention to avoid delay for fertility protection. N2: *“Whether or not she is able to fully accept it at that time, but you have to let her know to make her feel it. That stage should create an awareness for her to avoid her possibly like some egg preservation and missing that opportunity.”* N6: *“We are looking from the care team's point of view to be able to give them more attention to their needs in this area of reproduction and to give them some professional help from our point of view to minimize the regrets they have due to not knowing, you can choose not to, but hopefully you did have that information ingested before."*

Healthcare professionals should respect patients' rights to privacy and knowledge, strengthen communication with patients of reproductive age, enrich publicity and communication, and provide appropriate information support according to the patients' needs. N5: *“You can go and provide them with some counseling pathways and so on. One is the provision of information, which may pay more attention to the privacy aspect. …. .., if the patient has a detailed need to understand things, she can through these consulting ways.”* The Internet can be utilized to optimize access to reproductive information. It is possible to design decision-aid tools to facilitate patients' access to professional and scientific guidance, protect patients' privacy, and provide decision-making assistance. N15: *“Doing decision-aid type tools, or those brochures, to give to the patients to give them the idea of going to take a look at it. ”*

### Subtheme 2: Meso-systems level-emotional support from peers

Unlike healthcare professionals, peers can provide first-hand experiences of social coping, testimonies about treatment and recovery, and personal insights. N7: *“They can share some of their own experiences of coming over to them, including how to go to the doctor, how to go through the process … convenient access to reproductive-related knowledge, and when she encounters some problems, she can also continue to consult.”* Compared to healthcare professionals, peer communication is more likely to increase patients' empathy and trust, motivate them to participate in reproductive management, and serve as a social supplement to traditional health education model. N2: *“From the positive, I think it is definitely some of the successful cases, which some of the more optimized processes she has done, maybe we can learn from each other.”* Sharing success stories among patients can reduce feelings of isolation and anxiety and increase confidence.

However, peer supporters should be trained professionally before sharing and grouped according to patient characteristics to prevent blindly sharing negative experiences to avoid counterproductive situations. N1: *“This truly needs to be done. To be trained in these and not to talk nonsense, she will give positive things, and the level of knowledge and culture should be slightly higher, to match it.”* N12: *“If it is a matched patient, maybe some of the young ones together, maybe one will have a strong desire, the other one I think will be driven; there is an environmental role."*

## Theme 3: Reproductive management requires multi-system preparedness

Reproductive management involves the collaborative functioning of patients, the medical system, and social organizations, requiring multidimensional preparation to address complex needs.

### Subtheme 1: Micro-systems level-patient preparation

#### Patients need to enhance their awareness of fertility protection and take the initiative to communicate

Young patients need to increase their awareness of fertility protection, and understand that female patients can have more autonomy and flexibility in their reproductive choices. They must know that they can guarantee their possible future reproductive needs. N10: *" When you're young, you need to learn about fertility in your own way, such as looking at social propaganda, popular science, etc., you have to be willing to learn, save what you have first when you're at your best."*

At the same time, young patients should be aware of active communication, so that they can make healthcare professionals understand their personal reproductive needs. N10: *" You can take the initiative to raise this issue and ask us what methods we have, you may also want to know the price of the problem, including economic issues, etc., you need to ask, then we can understand clearly.”* N15: *“We also have some educational content now, and many people will watch it while waiting for medical treatment. At first they did not think of this problem, but after reading it, they may think that there is also this reproductive problem, …...*, *But the most important thing is you have to ask."*

#### Patients should improve their self-learning ability

Young patients should have the ability to actively learn and use various media to absorb reproductive-related information. N12: *“Now there are endless ways to obtain information, there are many multimedia platforms, and there are many kinds of live courses. Are patients willing to see and learn? If patients can learn on their own first, it will be convenient to communicate with us.”* N11:*"In the process of self-learning, patients can also put forward personalized needs and privacy protection needs, which is also convenient to establish a good communication bridge between doctors and patients."*

### Subtheme 2: Meso-systems level-medical preparation

#### Enhancement of the specialized knowledge base

Healthcare professionals are the most important means for patients to acquire specialized knowledge, and an inadequate knowledge base can affect communication and guidance. Nursing staff may lack a reproductive knowledge reserve, making communication about fertility protection difficult. N6: *“Nurses would like to care about, but they feel that they are a little bit lacking in their own professional knowledge, they do not know how to talk to her in depth about assisted reproductive technology; so they feel that their own ability would also be lacking.”*

(1) Reproductive education and innovative training

By attending specialized reproductive training, healthcare professionals can master reproductive knowledge to provide more comprehensive services to patients with reproductive needs, such as counseling on fertility protection measures and psychological support. N5: *“Healthcare professionals may want to be enriched with some knowledge in this area, and we have to prepare ourselves to …... reserve some knowledge …..."* It is important to enrich, consolidate and strengthen the practice content to develop a nursing team with specialized skills. For example, nurses can follow doctors' outpatient clinics, directly face patients to understand their needs and expectations, experience a wide variety of patients and disease cases, and then provide better-quality fertility counseling services in their work. N13: *“For example, go to see a certain patient in a specialized reproductive clinic, follow if you go to see the clinic and see what the fertility doctor would tell her. Kind of like we go and follow the lymphedema clinic now. It might be a little bit more impressive to go to an outpatient visit and see what questions the patient would have and how the doctor answered her."*

(2) Improving communication skills and establishing emotional exchange

Improving communication helps healthcare professionals establish trusting relationships with patients, increase their sense of security and trust, and understand their reproductive needs more accurately. N11: *“There is the fact that they have to be a little bit better at communicating …..., the emotional intelligence should also be slightly higher. Therefore, the speech-language communication piece is still important.”* Simulation exercises can be performed to develop patient communication skills. N8: *“How do you go with patients to communicate words, I think this face-to-face interactivity can let everyone participate in the process, we do a scenario simulation on site such."*

A few patients believe fertility is a private topic and are reluctant to discuss it. Nurses should communicate with patients in a timely manner to establish a bridge of trust conducive to understanding the patients’ inner thoughts and providing relevant help. N11: *“Because you cannot talk to her in a very straightforward manner, I do not think the patient will be able to talk to you about this matter very openly, and I think it is important to build up a bridge of trust between the patient and you."*

#### Increased awareness of reproductive management

Healthcare professionals often pay more attention to the treatment of diseases and ignore the reproductive needs of patients. Therefore, we should increase the value of reproductive issues for healthcare professionals, and cultivate their awareness, to assist patients in making rational and objective decisions.

Some young nurses neglect female patients' reproductive concerns because of their views on fertility. N11: *“Some of the younger nurses don't always go for the chat. Why? Because they do not pay attention to the reproductive piece, even if they are not married, that is, they do not think that much.”* Nurses' awareness of reproductive issues can be increased by incorporating reproductive assessments into specialty indicators for evaluation. N10: *“To raise awareness and incorporate it into our specialty quality indicators, there is definitely hope to raise it.”* Constructing a reproductive specialty clinic with trained specialty nurses that provides targeted counseling services would further increase the nursing team's awareness of reproductive management and comprehensive understanding of patients' reproductive needs. N11: *“For those who have an interest in this area. you can train one that is more specialized, you can specialize in one that is about fertility, then her specialty's definitely a little bit more specialized than your average nurse."*

### Subtheme 3: Macro-systems level-organizational readiness

#### Optimized medical procedures

Reproductive management involves multiple departments and specialties, and the fertility protection process is complex. This can be achieved by optimizing the management process, opening green channels, and simplifying the link to facilitate patients' access to medical consultation and treatment. Alleviating apprehension is an important aspect of organizational readiness. N9: *“The optimization of the process, helping them bridge reproductive-related and treatment processes that they need so that they do not have to think about it without worries; I think she will want to go."*

#### Multidisciplinary and optimized patient management model

Expert resources from various fields should be integrated, patients' reproductive problems should be analyzed and addressed from multiple perspectives, more comprehensive treatment plans should be provided, and the success rate of fertility treatment should be improved. N9: *“However, we think that there is a drawback that I do not have a way to unilaterally make an oncologist's decision; it is not a shared decision, and there's no way to go down and discuss together. Therefore, I'd say without an MDT, the patient is in a very confused state."*

Case management allows for the development of an individualized reproductive plan for each patient, ensuring the precision of care. Moreover, it enables ongoing follow-up and assessment, and, provides long-term support and guidance, which in turn improves the effectiveness of reproductive management. N15: *“We can think of it as case management, creating a file on you individually, how to track some of your information. Help you deal with your needs, how to refer you and all these things."*

#### Emphasis on continuous reproductive assessment and improvement of follow-up mechanisms

Patients diagnosed with cancer may not initially consider reproductive issues because of factors such as diagnostic shock. However, after treatment, such as surgery, the patient's feelings may change, and their understanding of the situation should be reassessed. N6: *"It's just possible that we've asked on routine admission …... when they're discharged, it is possible to care for those who haven't given feedback again and follow up with those people again.”*

Faster ward turnover and intense pressure on healthcare professionals affect reproductive management. The lack of a tracking mechanism for reproductive management leads to a lack of feedback regarding the patient's subsequent reproductive journey, making it impossible to follow the entire process. N11: *“Most of the time, it's because of the faster pace …. … "* Regular follow-up can provide patients with psychological support as well as personalized fertility guidance to help them achieve their reproductive goals safely and healthily. N3: *“I think the follow-up time should be at least 5∼10 years …. because many patients will choose endocrine therapy and will consider fertility only after the treatment is finished."*

Hospitals and social organizations can be united to build a whole-process management system to provide continuity of medical care services for patients as they require them, and integrate reproductive management into the entire process of health education for patients with breast cancer. N8: *“We wouldn't have a tracking mechanism,… …, do they need some follow-up continuity of care, and do they need us to intervene in it again. This can be linked up through social organizations in this process."*

## Discussion

We explored healthcare professionals' perspectives on the reproductive management of young women with breast cancer in China and identified three themes and eight subthemes through social-ecological systems. We categorized the management experience of healthcare professionals at the micro, meso and macro levels, and proposed improvement strategies from the patient, professional and organizational levels.

Reproductive wishes of young women with breast cancer are important factors in patient management. Younger age, childlessness, and the desire to have children are associated with a greater need for reproductive information.^22^ Healthcare professionals should dynamically evaluate patients’ situations, understanding, and psychological states, respect their personal wishes, be optimistic about fertility, and help patients understand the relationship between survival and fertility. Patients' privacy should be considered in this process, and private spaces should be provided as much as possible to allow patients to communicate openly. Family factors are the key obstacles to fertility.^23^ Professional reproductive guidance should be provided to the entire family, especially spouses, and family-based reproductive interventions should encourage them to make decisions that align with their preferences.

Most healthcare professionals indicated that patients were concerned about cancer survival and did not prioritize fertility. Our results showed that the key to empowering young patients to be proactive in learning about fertility, strengthening their learning abilities, and improving their health literacy lies in their efficient and easy access to specialized reproductive-related information. Currently, there are endless ways to obtain information, and the information available is uneven, making it easy for patients to lose their ability to discriminate. We believe personalized online oncological fertility decision aids and information leaflets can improve reproductive care.^24^ Young patients are eager learners, and professionals can enable them to become “educated” patients. The design of a simple and easy-to-use fertility decision aid or patient navigation tool can help patients enhance their self-learning skills while maintaining their privacy. Therefore, as healthcare professionals, we should create safe environments, provide professional and efficient tools, and enhance the awareness and ability of young patients to learn actively.

Our findings indicated that most professionals were less aware of the importance of discussing fertility. They can underestimate psychosocial issues beyond patients' survival,^25^^,^^26^ leading to a lack of concern about fertility and, therefore, omitting discussions about it. Moreover, healthcare professionals' attitudes are crucial for patients. The respondents in this study said that “the attitude of the oncologist is important at this time”. Therefore, it is necessary to increase professionals’ awareness of reproductive management through education.

Evidence suggests that health professionals do not engage in discussions about fertility after cancer,^27^^,^^28^ which may be related to a lack of knowledge.^29^ This is even more pronounced among nursing staff, with many rarely/never discussing reproductive-related issues with patients in clinical practice, fearing that they lack sufficient knowledge and competence to lead in-depth discussions.^30^^,^^31^ However, nurses play an important role on the oncology care team; they usually have longer contact with patients and families^32^ and conduct many reproductive discussions.^33^^,^^34^ Insufficient reproductive knowledge directly affects the provision of reproductive services to patients with cancer.^35^ Studies have shown that the greater nurses’ reproductive knowledge, the greater their willingness to provide reproductive counseling and health education^36^ and participate in reproductive discussions.^37^ Therefore, nursing staff should be trained to play a dominant role in the reproductive management of young patients and to draw on reproductive expertise to improve reproductive care. Ono et al.^38^ developed a web-based training program that covered the basics of reproductive medicine and supportive techniques for young patients with cancer, provided reproductive health-related information, and improved the knowledge and confidence of professionals regarding reproductive issues. Vadaparampil et al.^39^ designed a web-based training program (ENRICH) for reproductive health, primarily for oncology nurses, to improve their reproductive-related knowledge and behavior. After receiving adequate education, nursing staff successfully integrated reproductive management into clinical practice and better served young patients. Nursing administrators should pay increased attention to reproductive issues in female patients with cancer, develop continuing education courses for nurses, provide a knowledge-sharing platform, and construct and implement individualized reproductive training programs for nurses.^40^ The scope and breadth of nurses' roles should be optimized, an intervention system for nurses' reproductive education should be developed, an interdisciplinary learning platform should be built, regular and systematic training should be conducted, reproductive knowledge should be acquired using case sharing, and salons should be enriched so that nurses have sufficient knowledge, confidence, and competence to conduct reproductive discussions with patient with cancer and their families.

Our findings showed that, when young patients faced reproductive problems, most did not readily acknowledge their concerns to their doctors and did not have the opportunity or sufficient time to request medical advice. Most professionals remained silent on reproductive issues and, lacked communication mechanisms. Enhanced communication is the key to a successful reproductive supportive care program.^41^ Healthcare professionals should listen attentively to patients' concerns, pay attention to patients' reproductive information preferences, and provide empathetic feedback.^42^ Lien et al.^43^ developed a decision-making model for nurse oncology reproductive counseling that included four stages: perceiving patient needs, triggering self-emotions, empathizing with the patient, and reflecting on their role. Perceiving the patient's needs is the first step in initiating a reproductive counseling session, indicating the importance of communication. Therefore, special attention should be paid to communication skills when educating professionals. Communication skills training is specifically designed for the ENRICH training course,^39^ and the educational program Fex-Talk improves the communication skills of nursing staff through role-playing.^34^

Time is the most common institutional barrier. For newly diagnosed patients, the concept of fertility takes some time to accept, but the window of time left for intervention is narrow; thus, they must receive and understand information and make decisions about fertility quickly. In this case, patients relied mostly on their oncologists' advice and guidance. For healthcare professionals, overwhelming clinical workloads, busy nurse shifts, and lengthy consultation and referral processes are time-related barriers.^30^ Therefore, at the organizational level, there is a need to manage the consultation and referral processes, promote inter-professional cooperation, maximize the integration of medical resources, and improve the overall efficiency of medical services. Moreover, simple decision aids should be developed to help healthcare professionals familiarize themselves with the processes, facilitate communication, and efficiently assist patients in making decisions within a limited amount of time.

At the organizational level, the competence of clinical professionals can be enhanced through continuing education.^44^ Multidisciplinary knowledge alliances facilitate the sharing of different types of expertise. A survey in China revealed that most people have a positive attitude toward interdisciplinary collaboration between oncologists and reproductive specialists in oncological reproductive practices, supporting the establishment of a platform for oncology-reproductive medicine multidisciplinary collaboration.^45^ Reproductive counseling and educational interventions in multidisciplinary collaborative models can improve reproductive knowledge and alleviate reproductive concerns in oncology patients^46^; however, the current operation of multidisciplinary collaboration in China still suffers from insufficient human resources and a lack of adequate management.^47^ Thus, multidisciplinary collaboration system in oncology needs further improvement.

Zwingerman et al.^48^ used telemedicine and oncology reproductive navigators to provide fertility protection information and reproductive counseling to reproductive-aged oncology patients in remote areas. During a year-long pilot study, providers and patients benefited from the support provided through education and care navigation, and nurse navigators became champions of oncological reproduction. Nurse navigators have been shown to increase the likelihood of reproductive counseling and patient satisfaction in reproductive management programs for young patients.^49^, ^50^, ^51^ The effectiveness of nurses’ involvement in oncological reproductive care has been confirmed in previous research.^52^ Our research also suggests that the role of nursing staff in multidisciplinary collaboration in reproductive management is worthy of further exploration and practice. Nurse-led oncofertility counseling clinics could be implemented to increase professional confidence and patient trust through the many roles of nurses.^53^ The experience of patient navigators in oncological reproductive management has also been favorable.^54^ Patient navigators can provide psychological guidance and emotional experience and encourage positive results. At the organizational level, standardized navigation training, assessment, and implementation protocols are necessary to ensure the quality of oncologic guides.^55^ Moreover, it is necessary to coordinate the development of support resources and patient navigation tools for the effective reproductive management of young patients.

### Limitations

The participants in this study were healthcare professionals from one cancer center. Therefore, the representation and generalizability of the sample are limited.

Since this study only used content analysis to describe professionals' experiences and perspectives in reproductive management for young women with breast cancer, we hope to use phenomenological research to deeply explore the meanings behind these experiences in future study, including professionals' inner experiences when making clinical decisions, personal or ethical challenges they face when providing fertility counseling, and the facilitating and hindering factors behind these experiences. We also aim to explore whether these experiences change with increasing personal capacity or over time in future study.

### Recommendation

The inclusion of healthcare professionals from multiple centers is recommended to expand the representation and generalizability of the sample. More multidisciplinary specialists in reproductive issues should be interviewed to ensure data diversity.

### Implications for nursing research and education

It is necessary to design various evidence-based fertility educational programs to provide a theoretical and practical learning platform for nursing staff. Enhancing all nurses' cancer reproductive consulting abilities and training reproductive cancer specialist nurses to participate in multidisciplinary collaborations is essential. In addition, future research could strengthen researches on fertility decision aids and the characteristics of reproductive navigators that are suitable for China's unique conditions.

## Conclusions

This study used the social-ecological system theory to identify barriers and strategies at different levels in the reproductive management of young women with breast cancer, and to promote the reproductive management of young patients by healthcare professionals at the micro-, meso-, and macrosystem levels. From the professional perspective, we should respect the patient's will, consider family decisions, and strengthen learning abilities. Meanwhile, we should also improve the reproductive management awareness of healthcare professionals, their professional learning ability and their cancer reproductive consultation and communication abilities. At the organizational level, social resources can be integrated to provide multidisciplinary information and multi-dimensional emotional support to promote smooth reproductive decision-making in young patients with breast cancer and to improve their quality of life.

## CRediT authorship contribution statement

**Jiajia Qiu**: Conceptualization (Lead); funding acquisition (Lead); methodology (Equal); project administration (Equal); interview (Equal); supervision (Lead); visualization (Equal); writing – original draft (Equal); writing – review & editing (Lead). **Jing Li**: Methodology (Equal); data curation (Equal); interview (Equal); project administration (Equal); resources (Equal); analysis (Equal); writing – review & editing (Equal). **Lichen Tan**g: Data curation (Equal); resources (Equal). **Ping Li**: Data curation (Equal); resources (Equal). **Mingxuan Cai**: Data curation (Equal); analysis (Equal); writing – original draft (Equal). **Chenxi Zhu**: Data curation (Equal); analysis (Equal); writing – original draft (Equal). All authors have read and approved the final manuscript.

## Ethic statements

This study was approved by the Scientific and Ethical Committee of the Shanghai Cancer Center, Fudan University (IRB No. 2205254-Exp1) and was conducted in accordance with the 1964 Helsinki Declaration and its later amendments or comparable ethical standards. All participants provided written informed consent.

## Data availability statement

The data that support the findings of this study are available from the corresponding author, JQ, upon reasonable request.

## Declaration of generative AI and AI-assisted technologies in the writing process

No AI tools/services were used during the preparation of this work.

## Funding

This work was supported by 10.13039/100017950Shanghai Municipal Health Commission (Special Clinical Research of Health industry). The funders had no role in considering the study design or in the collection, analysis, interpretation of data, writing of the report, or decision to submit the article for publication.

## Declaration of competing interest

The authors declare no conflict of interest.
